# Quantitative detection of Staphylococcus aureus, Streptococcus pneumoniae and Haemophilus influenzae in patients with new influenza A (H1N1)/2009 and influenza A/2010 virus infection

**DOI:** 10.3205/dgkh000249

**Published:** 2015-04-15

**Authors:** Firouzeh Safaeyan, Mohammad Reza Nahaei, Sirus Jedary Seifi, Hossein Samadi Kafil, Javid Sadeghi

**Affiliations:** 1 Tuberculosis & Lung Disease Research Center, Tabriz University of Medical Sciences, Tabriz, Iran; 2 Department of Medical Microbiology, Faculty of Medicine, Tabriz University of Medical Sciences, Tabriz, Iran; 3 Drug Applied Research Center, Tabriz University of Medical Sciences, Tabriz, Iran

**Keywords:** S. aureus, S. pneumoniae, H. influenzae, bacterial colonization, H1N1, influenza A

## Abstract

**Introduction: **Viral influenza is a seasonal infection associated with significant morbidity and mortality. In the United States more than 35,000 deaths and 200,000 hospitalizations are recorded annually due to influenza. Secondary bacterial infections or co-infections associated with cases of influenza are a leading cause of severe morbidity and mortality, especially among high-risk groups such as the elderly and young children.

**Aim:** The aim of the present study was the quantitative detection of *S. aureus, S. pneumoniae *and* H. influenzae* in a group of patients with seasonal influenza A, influenza A (*H1N1*) pandemic 2009, and patients with symptoms of respiratory infection, but the negative for *H1N1* serving as control group.

**Method:** In total, 625 patients suspected respiratory infection from April 2009 to April 2010 were studied. There were 58 patients with influenza A *H1N1* and 567 patients negative for influenza A *H1N1*. From November 2010 to February 2011, 158 patients with respiratory symptoms were analyzed for seasonal influenza A. There were 25 patients with seasonal influenza A. To check the colonization status among the healthy individuals 62 healthy persons were further investigated. Individual were screened in parallel. The choices of special genes were amplified from clinical specimens using real-time PCR with a cutoff of 10^4^ CFU/mL to differentiate colonization from infection in respiratory tract.

**Results:** *S. aureus, S. pneumoniae *and* H. influenzae* were detected in 12%, 26% and 33% of patients with *H1N1*, while the corresponding figures were 9%, 19%, and 31% for *H1N1* negative patients. Among patients with seasonal influenza A 12% *S. aureus,* 24% *S. pneumoniae*, and 32% *H. influenzae* co-infections were detected, while influenza negative control group yielded 5% *S. aureus*, 11% *S. pneumoniae*, and 10% *H. influenzae*, respectively.

**Conclusion:** The results of this study indicated that the serotype of pandemic* H1N1* 2009 did not increase incidence of secondary infection with *S. aureus, S. pneumoniae* and *H. influenzae*. Quantitative detection of secondary bacterial infection by QR-PCR can help us for distinguishing colonization from infection and controlling misuse of antibiotics and bacterial drug resistances.

## Zusammenfassung

**Einleitung:** Die Virusgrippe ist eine saisonale Infektionskrankheit, die mit ausgeprägterer Morbidität und Mortalität einhergeht. In den USA werden jährlich mehr als 35.000 Todesfälle und 200.000 Krankenhausbehandlungen erfasst. Die mit der viralen Primärinfektion assoziierte bakterielle Superinfektion oder Ko-Infektion verursacht schwere Krankheitsverläufe speziell bei Hochrisikogruppen wie alten Menschen und Kleinkindern.

**Zielsetzung:** Die Zielsetzung der Studie bestand in der quantitativen Bestimmung von *S. aureus, S. pneumoniae* and *H. influenzae* bei Patienten mit saisonaler Influenza A bzw. pandemischer Influenza A (*H1N1*) und Symptomen respiratorischer Infektionen, im Vergleich zu je einer Kontrollgruppe.

**Methode:** Insgesamt wurden von April 2009 bis April 2010 625 Patienten mit Verdacht auf eine respiratorische Infektion untersucht, davon 58 Patienten mit Nachweis von Influenza A (*H1N1*). Vom November 2010 bis zum Februar 2011 wurden 158 Patienten mit respiratorischen Symptomen auf das Vorkommen der saisonalen Influenza A untersucht, davon erwiesen sich 25 als positiv. Zur Ermittlung der bakteriellen Kolonisation wurden parallel 62 gesunde Personen untersucht (Kontrollgruppe). Bei der verwendeten Real-time PCR wurde als Cutoff zur Unterscheidung von Koloniation und Infektion im Respirationstrakt 10^4^ CFU/ml eingeführt.

**Ergebnisse: ***S. aureus, S. pneumoniae *und *H. influenzae* wurden bei 12%, 26% bzw. 33% der Patienten mit Nachweis von Influenzavirus A (*H1N1*) gefunden; die Häufigkeit in der Kontrollgruppe betrug 9%, 19% bzw. 31%. Bei der saisonalen Influenza A waren bei 12%, 24% bzw. 32% die Erreger nachweisbar, in der parallelen Kontrollgruppe bei 5%, 11% bzw. 10%.

**Schlussfolgerung**: Die Ergebnisse zeigen, dass der Serotyp der pandemischen Influenza A (*H1N1*) die Inzidenz der bakteriellen Superinfektion für die drei untersuchten Bakterienspecies nicht erhöht hat. Die quantitative Detektion einer sekundären bakteriellen Infektion mittels Real-time PCR ist geeignet, zwischen Kolonisation und Infektion zu unterscheiden und damit einer missbräuchlichen Anwendung von Antibiotika vorzubeugen.

## 1 Introduction

The novel influenza A (*H1N1*) virus pandemic, emerged in the spring of 2009 as a consequence of interactions between human, avian and swine influenza viruses. Because of a new type of the Haemagglutinin, this pandemic (*H1N1*) strain rapidly spread worldwide and recorded as the first pandemic of influenza in 21^th^ century [1], [2]. The 1918 influenza virus pandemic resulted in approximately 50 million deaths worldwide, a majority of which were associated with secondary bacterial infections [3]. Secondary bacterial infections were also detected in yearly epidemics and pandemics. These infections are seen in 30% of patients with seasonal influenza [4], [5]. Secondary bacterial infections that follow infection with influenza virus result in considerable morbidity and mortality in young children, the elderly, and immunocompromised individuals and may also significantly increase mortality in normal healthy adults during influenza pandemics [6]. Recently, analysis of 34 autopsy cases of individuals who died from the 2009 *H1N1* pandemic influenza virus showed that over half displayed signs of secondary bacterial infections by both postmortem lung culture and histological evaluation [7].

Bacterial complications are more common during pandemics [8]. The most frequently detected pathogens are *S. aureus, S. pneumoniae, S. pyogenes, *and* H. influenzae.* These bacteria colonize in pharynx and nasopharynx of human especially in children. They could change from not having symptoms to invasive form following viral infection [5], [9], and their number is more during the time of respiratory infection than carriers [10].

The mechanism of secondary bacterial infection is augmented by attachment and colonization of bacteria during viral infections due to injury of respiratory epithelium. Major drawbacks of this mechanism include not only the increased risk of physical and functional alterations in respiratory tracts, but also change inflammatory responses which leads to enhanced replication of viruses and conditions for secondary bacterial infections are prepared. Protease secretion by some of bacteria colonized in the upper respiratory tracts increases virus virulence [11], [12]. HA cleavage is necessary for virus cell entry by receptor-mediated endocytosis. Based on studies with staphylokinase, streptokinase, and other activating host proteases, such as kallikrein, urokinase, thrombin, and plasmin it is believed that microbial proteases can potentiate the cleavage activation of influenza virus by mechanisms other than direct cleavage of the HA. Such mechanisms could increase inflammation or destroy endogenous protease inhibitors [13]. In addition, it is possible that microbial proteases could contribute to host protease activity by increasing inflammation or destroying endogenous protease inhibitors.

In this study, we aimed to investigate quantitative burden of secondary bacterial infections in patients with influenza A (*H1N1*) pandemic 2009 (between April 2009 until April 2010) and in seasonal influenza A (between November 2010 until February 2011) in Northwest of Iran. 

## 2 Material and methods

### 2.1 Sample collection 

Nasopharyngeal samples for viral testing were collected from inpatients and outpatients from health centers, EMAM REZA, SINA, and children’s hospitals using rayon swabs and placed into 3 mL of Universal Viral Transport Medium (VTM). Microbiological testing was performed on a second sample from the nasopharynx of patients for microbiological testing. After sampling, swabs were kept in sterile tubes containing NaCl (0.8%) and both samples stored at –80°C until used.

From April 2009 to April 2010, 625 samples from 625 individual patients with acute onset of respiratory symptoms accompanied by fever and a recent travel history to countries with sustained human-to-human transmission of pandemic *H1N1* influenza were analyzed with the novel pandemic *H1N1* influenza real-time PCR assay. From November 2010 to February 2011, 158 samples collected from patients with respiratory symptoms infection.

### 2.2 Pandemic H1N1 influenza-specific real-time RT-PCR assays

Real-time reverse transcriptase-polymerase chain reaction (rRT-PCR) with subtype-specific primers for detection of influenza A (*H1N1*) pdm09 was used. RNA was extracted using QIAamp Virus RNA Mini Kit (Qiagen) according to the manufacture’s protocol. The RNA was eluted to a final volume of 100 μL. Water was used as negative control and an influenza virus isolate, A/Stockholm2/2009* H1N1*, was used as positive control for each extraction. 

The primers used were 5’GGC TGC TTT GAA TTT TAC CAC AA 3’ and 5’TTT GGG TAG TCA TAA GTC CCA TTT T 3’, amplifying the hemagglutinin gene. The probe used was 5’-FAM-TGC GAT AAC ACG TGC ATG GAA AGT GTC-TAMRA-3’. For the PCR, the SuperScript III Platinum One-Step Quantitative RT-PCR system was used (Invitrogen Corporation, Carlsbad, CA, USA). The PCR program used was reverse transcription for 15 minutes at 50°C followed by 2 minutes at 95°C, 45 cycles of 95°C for 5 seconds, 60°C for 60 seconds, and 40°C for 30 seconds using the LightCycler 480 (Roche Diagnostics GmbH, Mannheim, Germany). A threshold cycle (CT) of ≤40 together with a sigmoid fluorescence curve was needed for the result to be considered positive [14].

### 2.3 RT- PCR assays for general influenza A (MA) 

144 suspected cases were analyzed for influenza A. Published real-time PCR primers targeting the matrix (MA) gene of influenza A virus were used. A PCR reaction (One-step RT-PCR kit, Qiagen) of 25 µL for the matrix assay contained 5 µL of RNA extract, 1x reaction buffer, 400 µM of each dNTP, 40 ng/µL bovine serum albumin, 400 nM of primer M_InfA F (AAGACCAATCCTGTCACCTCTGA; GenBank Accession number CY038773(175-197), 400 nM of primer M-_InfA-R (CAAAGCGTCTACGCTGCAGTCC;nt 269-248), 200 nM of probe M_InfA TM (FAM-TTTGTGTTCACGCTCACCGT-BBQ; nt 215-234) and 1 µL of enzyme mix. The following conditions were met: 30 min at 50°C; 15 min at 95°C; 45 cycles of 15 s at 94°C; and 30 s at 60°C [15].

### 2.4 Viral culture

Confluent monolayers of MDCK cells grown in Dulbecco’s modified Eagle’s medium were inoculated with fresh respiratory specimens and monitored daily for cytopathic effect.

Immunofluorescence testing by using the monoclonal antibody against nucleoprotein was performed when cytopathic effect was identified or 8 days post inoculation if no cytopathic effect was observed [16]. 

### 2.5 Patient panel

Studied patients consisted of 4 panel which were chosen as described below:

Panel A: were 58 (36 females and 12 males) with average age of 31 years; Patients being positive for influenza A (*H1N1*) viral infection in 2009. 

Panel B: were 58 (39 females and 19 males) having average age of 48 years; Patients being negative for A (*H1N1*) viral infection in 2009 but had symptoms of respiratory infections. 

Panel C: were 25 (12 females and 13 males) with average age of 52 years; Patients being positive for seasonal influenza A virus in 2010. 

Panel D: were 62 healthy persons (38 females and 24 males) with average age of 30 years, all being confirmed by physician diagnosis and chest radiography results of not having respiratory infection, having negative CRP and for which ESR, CBC and WBC were normal. None of these individuals had fever, cough, sneezing or other typical symptoms of respiratory infections. 

### 2.6 DNA extraction

Respiratory tract samples were first centrifuged for 10 min with 1,500 RPM centrifuge, then the supernatant solution was removed and samples were washed twice with PBS and resuspended in 500 mL of lysis buffer (Buffer AL, Qiagen, Crawley, UK) to preserve the nucleic acids [17]. Platelets were treated with 10 mL of an in-house lysis solution to lyse bacterial cells. This stock solution consisted of 10 mL of filter-sterilized buffer 1 (20 mM Tris (pH= 8), 2 mM EDTA (Sigma) 1.2% Triton X- 100), 500 mg of lysozyme (Sigma, Poole, UK), and 1,000 U of Lysostaphin (Sigma, Poole, UK). The applied genomic DNA extraction kit was provided from Qiagen, Crawley, UK [18].

### 2.7 Quantitative and qualitative PCR

Special *nuc* gene of *S. aureus *strains which is in charge of nuclease production. This enzyme is resistant to heat, is extracellular and an enzyme that catalyzes the hydrolysis of both DNA and RNA. *ply* gene producing pneumolysin which is the specific toxin of pneumococcus and is specially produced by clinical isolates. The nucleotide sequence of the forward primer was 5’-AGCGATAGC TTT CTC CAA GTG G-3’ (position 531 to 552), and the sequence of the reverse primer was 5’-CTT AGC CAA CAA ATC GTT TAC CG-3’ (position 583 to 605) with an amplicon of 75 base pairs.

*frdb* is a housekeeping gene related to fumarate reductase iron-sulfur gene B (*frdb*) was chosen. This gene has low homology with other *Haemophilus *spp. The nucleotide sequence of the forward primer was 5’-ATC GAA AGT TTA GAG GCA A-3’ (position 328 to 348), whereas the sequence of the reverse primer was 5’-TTC TTT CGA TGG ATG TGG TT-3’ (position 392 to 412). The amplicon was 84 base pairs. All of the chosen genes were designated by an evaluation of all available sequences in GenBank and it was tried to be completely specific. *frdb* and *ply* primers were chosen according to former studies [19] and *nuc* primer sequence was as below. Sequence of F primer was 5’-GCGATTGATGGTGATACGGTT-3’ (position 48 to 70) and sequence of R primer was 5’-GCCAAGCCTTGACGAACTAAAG-3’ (position 303 to 328). The amplicon was 280 base pairs [20]. The presence of amplifiable DNA in all extracts was verified by amplification of human house-keeping genes 18S-RNA. Forward primer 5’-TTCTGCCCTATCAACTTTCG-3’ reverse primer 5’-GATGTGGTAGCCGTTTCTCA-3’ [21] and amplicon size was 112 base pairs.

 The RQ-PCR assay was performed in Rotor-Gene 3000 (Corbett Research, Mortlake, Sydney, Australia) and analyzed by using a software program from Real-Time Analysis Software (Corbett Research). The real-time PCR amplifications were performed in 20 µL reactions containing 2X QantiTect SYBR Green PCR master mix (Takara, China), which includes HotStarTaq DNA Polymerase, QuantiTect SYBR Green PCR Buffer, 2.5 mmol/mL MgCl_2_, deoxyribonucleotide triphosphate (dNTP) mix, and fluorescent dyes, RNase-free H_2_O (SIGMA; Sigma-Aldrich,Germany), 0.6 mmol/mL primer (cinagen, Iran), and 2 µL of the respective template DNA dilution. All of the experiments were performed twice. The concentration of DNA was measured by NanoDrop ND-1000 Spectrophotometer (NanoDrop Technologies, Wilmington, DE).

### 2.8 RQ-PCR protocol

RQ-PCR assay for *S. aureus*, *S. pneumoniae* and *H. influenzae* was optimized to the initial activation step of 95ºC for 15 min, followed by 40 cycles of denaturation at 94°C for 20 s, annealing at 50°C of 60 s for *nuc*, 60°C for *ply* and 50°C for *frdb* extension at 72°C for 25 second followed by a final extension step at 65°C for 30 s.

### 2.9 Standard curves for positive controls 

*S. aureus* ATCC 29213, *S. pneumoniae* ATCC 6305 and *H. influenzae* ATCC 9006 were used as positive controls in drawing the standard curve. Positive controls containing known concentrations of template were used for establishing correlation curves between bacterial concentrations and cycle threshold (CT) values in the RQ-PCR. Two different serial dilution schemes were made: one for viable counts and CFU/mL calculation, and one for DNA quantification with RQ-PCR. A dense suspension of bacteria grown on agar plates was inoculated in phosphate-buffered saline, representing a bacterial concentration of approximately 10^8^ CFU/mL. The concentration of DNA corresponding to 10^8^ CFU/mL was determined spectrophotometrically with the NanoDrop ND-1000 Spectrophotometer (NanoDrop Technologies, Wilmington, DE). Starting from this concentration, a 10-fold serial dilution scheme ranging between 10^8^ and 10^3^ CFU/mL was prepared. Number of CFUs was determined by plating 10 µL of each dilution step onto agar plates and incubating them at 37°C in 5% CO_2_ overnight. The number of colonies was counted after 24 h. Two hundred µL of the highest concentration was used for DNA extraction. Genomic equivalents (GEQ) per mL were calculated by use of the following website: http://cels.uri.edu/gsc/cndna.html

### 2.10 Statistical analysis

Statistical analysis was performed by t-test between mentioned groups. The number of detected patients carrying mentioned bacteria wasn’t significant in comparison to other patients, but was significant compared to the control group (P-value <0.05).

## 3 Results

### 3.1 Viral findings

During the pandemic 2009, 625 samples were investigated and 58 (9%) samples were positive for influenza A (*H1N1*) by real-time PCR and viral culture assay. Thirty-six samples were obtained from female, and 12 from male patients with an average age of 31 years. Eleven (19%) samples belonged to patients older than 65 years. Thereof, 3 (5%) patients had cancer and immune suppression, 1 patient was pregnant at age 30 years. Twelve (21%) patients were under 5 years old. Thirty-three (57%) patients were hospitalized. 567 (91%) patients had negative results for influenza A (*H1N1*). From patients with symptoms of respiratory infections, but negative for influenza A (*H1N1*), 58 were selected for further study (consisted of 58 patients, 39 females and 19 males) having an average age of 48 years. 22 patients were older than 65 years, and 10 patients younger than 5 years. Twenty-one patients were hospitalized.

From November 2010 until February 2011, during seasonal influenza A, a total of 25 patients were confirmed for influenza A with real-time PCR and viral culture assay, thereof 12 females and 13 males, with an average age of 52 years. Five patients were older than 65 years, and 5 patients younger than 5 years. None of the patients were hospitalized.

### 3.2 Bacterial findings

#### 3.2.1 Sensitivity and amplification efficiency of the RQ-PCR 

The concentration of DNA corresponding to *S. pneumoniae* with 10^8^ CFU/mL was approximately 40 ng/µL, for *H. influenzae* with 10^8^ CFU/mL was 93 ng/µL and for *S. aureus* with 10^7^ CFU/mL was 30.3 ng/µL. GEQ per milliliter were calculated for the three species and were *S. pneumoniae*: 1.8×10^9^, *H. influenzae*: 4.7×10^9^, and *S. aureus*: 9.6×10^12^ GEQ/mL. 

**Figure 1:**
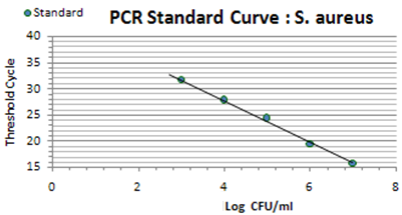
Standard curve showing consistency in CT values between serial dilutions (*S. aureus*)

**Figure 2:**
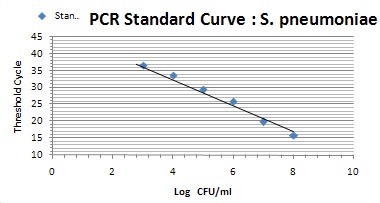
Standard curve showing consistency in CT values between serial dilutions (*S. pneumoniae*)

**Figure 3:**
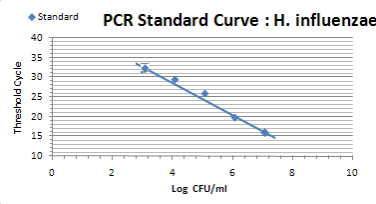
Standard curve showing consistency in CT values between serial dilutions (*H. influenzae*)

The relation of the CT standard curve with each of standard concentrations is shown in Table 1 (Tab. 1) (Figure 1 (Fig. 1), Figure 2 (Fig. 2), Figure 3 (Fig. 3)).

#### 3.2.2 Sensitivity of real-time PCR

Tenfold serial dilution from a concentration of 10^8^ CFU/mL of *S. pneumoniae* and *H. influenzae* and 10^7^ CFU/mL of *S. aureus* bacteria were used to construct standard curves for determination of the sensitivity of the real-time PCR assay. 

Prepared standard curve of serial dilution for S.* aureus, S. pneumoniae *and* H. influenzae* bacteria showed the sensitivity of PCR, which were 10^3^ bacteria in this study. The amount of *S. aureus* DNA is demonstrated by 15 to 28 CT for 10^7^ to 10^3^ bacteria. The amount of *S. pneumoniae* DNA, which was detectable in this study, was demonstrated by 15 to 33 CT for 10^8^ to 10^3^ bacteria. Finally the amount of detectable DNA of *H. influenzae* bacteria was demonstrated by 15 to 31 CT for 10^8^ to 10^3^ bacteria.

#### 3.2.3 Correlation, coefficient and amplification efficiency

The amplification efficiency was determined by plotting cycle threshold values against the DNA copy number and calculating the correlation coefficient. The calculated amounts of study bacteria are demonstrated for *S. aureus* (E=79.5%, R2=0.998), *S. pneumoniae* (E=71.6%, R2=0.991), and *H. influenzae* (E=83.7%, R2=0.998).

#### 3.2.4 Specificity of real-time PCR

Specificity of real-time PCR was determined by the ability of detecting different bacterial species. The bacteria were used *S. mitis ATCC 15912, S. pneumoniae ATCC 33400 *and* E. coli ATCC 33930*. Also the specificity of the PCR is temperature dependent; the annealing temperature had to be optimized for the respective microorganisms. Melting curve analysis is another important method for determining the specificity of the PCR products. Accepted temperature range for the melting curve analysis was 79.9–81.2ºC for *S. pneumoniae*, 76.0–77.2ºC for *H. influenzae*, and 81.5–82.5ºC for *S. aureus* 81.5–82ºC. *ply* primer is totally specific in detection of *S. pneumoniae* at annealing temperature of 60ºC–65ºC, whereas other strains of *α hemolytic streptococcus* of 10^8^ concentrations have higher CT values in this temp. Also, it was indicated that *frdb* primer is specific for *H. influenzae* at temperature of 50ºC and some strains of *H. parainfluenzae* have CT values lower than calculated in this research. Furthermore, it was observed that *nuc* was specific at 58ºC.

#### 3.2.5 Reproducibility 

**Figure 4:**
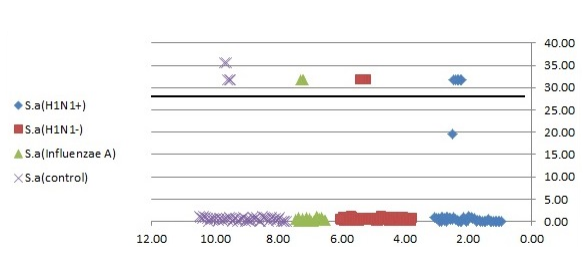
Association of nasopharyngeal bacterial colonization of *S. aureus* with CT

**Figure 5:**
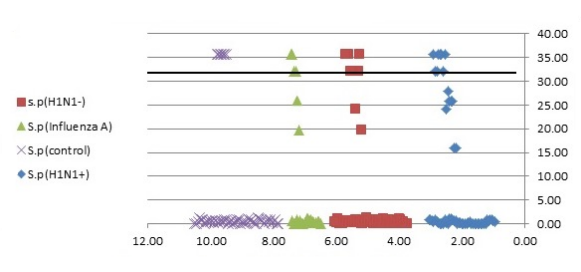
Association of nasopharyngeal bacterial colonization of *S. pneumoniae *with CT

**Figure 6:**
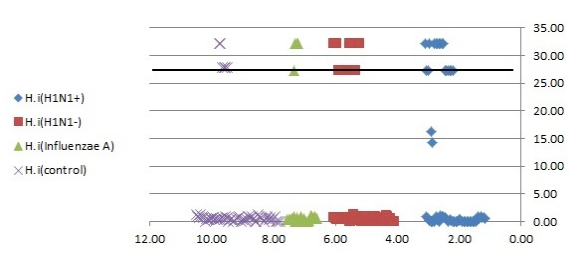
Association of nasopharyngeal bacterial colonization of *H. influenzae* with CT

Serial dilutions of positive control samples were performed three times (triplicate) for each experiment. A slight inter-assay variation in CT values in the dilution series of the positive controls could be observed. Few and periodic changes of CT for serial dilution of positive control are demonstrated in Figure 4 (Fig. 4), Figure 5 (Fig. 5), Figure 6 (Fig. 6). These numbers show the relationship between different concentrations of the same DNA run 3 times on different occasions. The intra-assay variation was very low, as determined by running duplicates of the same samples in the same run. Difference of CT in different serial dilutions of DNA was gained after about 3 to 4 cycles, which showed that PCR is repeatable.

#### 3.2.6 Bacterial quantitative assay

According to the amount of CT in the control group, the cut-off for all 3 bacteria was drawn on the CT which demonstrated 10^4^ CFU/mL bacteria for *S. aureus*, CT=28, *S. pneumoniae,* CT=31, and *H. influenzae*, CT=27.

Patients with CT<cut-off with high bacteria were not detected in Influenza A group. Five (*H1N1*) positive patients older than 65 years, and three (*H1N1*) positive patients under the age of 5 years had *S. pneumoniae* CT<cut-off. Two (*H1N1*) positive patients younger 5 years had *S. aureus* CT<cut-off. Three (*H1N1*) positive patients younger than 5 years had *H. influenzae* CT<cut-off. Two (*H1N1*) negative patients older than 45 years (ICU hospitalized and treated with ventilator) and one patients younger than 5 years had *S. pneumoniae* CT<cut-off. All patients with CT<cut-off for all three bacteria were hospitalized (Table 2 (Tab. 2), Table 3 (Tab. 3)).

## 4 Discussion

In spite of progress in diagnostic microbiology, difficulties in the detection and differentiation of colonization in infections remain to be resolved. In this study consisting of samples from patients with influenza A and the pandemic *strain H1N1*, RQ-PCR substantially improved significant pathogen detection and quantitative. Epidemiologic studies show symmetry between peaks of influenza and bacterial pneumonia infections, noticing that patients with influenza infection are assumed to be an active source of secondary bacterial infections; rapid detection and isolation of these individuals from the public are the ways for preventing secondary bacterial infections [15]. Because bacteria that participated in secondary infections are mostly known as microbial flora, quantitative detection of these bacteria seems to be essential. The presence of commensals may impair both identification and quantification of potential pathogens, leading to false-negative cultures or quantitative underestimation of relevant pathogens. Both respiratory cultures and blood cultures have low sensitivities for the detection of respiratory pathogens. Usual methods of blood and sputum culture are time consuming and have low sensitivities for the detection of respiratory pathogens. False positive and negative results are common in these methods. In the method of RT-PCR false positive results caused by colonization of microbial flora is prevented by use of proper cut off. Adjusting cut off to low levels of infection is performed during pandemics and the spread of pneumonia in order to increase real-time PCR’s sensitivity [22], [23]. Pertaining to the quantitative detection of bacteria, antibiotic treatment with drugs active against the causative agents before sampling, cause decrease in appearance of resistant bacteria and reducing the usage of broad spectrum antibiotics and new generations’ side effects [2]. Detection of secondary bacterial infections especially during pandemics in which lots of people are infected causes saving governmental economic costs. However, it may decrease the use of broad-spectrum antibiotics, which are usually expensive, and reduces the hospitalization period of patient in hospitals. 

In our study, we assessed 62 healthy individuals as controls. These subjects were divided in two groups, the first group consisted of individuals younger than 4 years, and the second group of individuals older than 4 years. Hence, the first group consisted of 20 children with an average age of 2.3 years, and the second group of 42 individuals with an average age of 43.4 years. In the first control group, 60% of children gave positive results in real-time PCR assay for all three bacteria, 2 positive for *S. aureus,* 3 positive for *S. pneumoniae*, and 5 positive for *H. influenza*e. In the second group, 14.3% of the included subjects gave positive results. One positive for *S. aureus*, 4 positive for *S. pneumoniae*, and 1 positive for *H. influenza*e. Three bacterial load calculated by real-time PCR. Both groups were below 10,000 CFU/mL. 

However, there results needs to be evaluated on larger population. The main reason for selecting these pathogens was in particular their importance as global health problems, as super-infections due to *S. aureus* following influenza are an increasing concern [24]. Appearance of USA400 and USA300 clones and the predominant community-associated methicillin-resistant *Staphylococcus aureus* (CA-MRSA) often contain Panton-Valentine leukocidin (PVL) genes and, more frequently, cause necrotic and fulminant pneumonia and shock, especially in children whit influenza [25]. The impact of interventions against influenza and *S. pneumoniae*, the bacterium that most frequently is the cause of community-acquired pneumonia, can be seen in directed vaccination studies, where, in at-risk populations, hospitalizations were reduced by 18%–52% and mortality was reduced by 35%–61% [26].

A single study conducted in 1945 showed that infection with both influenza virus and *H. influenzae* killed mice at doses that were sublethal when either agent was administered alone [6].

The incidence of secondary bacterial infection varies with the age and infirmity of the patient and the strain of infecting influenza virus: while individuals in recognizing risk groups have the highest risk of bacterial infections, younger patients and children also have a significantly increased risk. Indeed, a study published by Rothberg et al. [27] investigating complications associated with influenza in different age groups confirmed the selected CT<cut-off for patients younger than 4 years of age. Previous studies have shown higher numbers of bacterial samples taken and culture positivity in patients with influenza A than patients with influenza A (*H1N1*) pandemic 2009 (*P*<0.0001 and *P*=0.01, respectively). In our study, patients diagnosed with all three bacteria were almost equal in both groups of patients, influenza A and influenza A (*H1N1*) pandemic 2009, but our study patients with CT<cut-off in the influenza A group was not found [14]. In a study by Palacios et al. [28] which was performed on 199 *H1N1* patients by the method of Tag-PCR on nasopharynx samples, the detection rate of 3 bacteria (*S. aureus, S. pneumoniae, *and* H. influenzae*) were 52.3%, 35.5% and 20.6%, respectively, in patients with influenza A (*H1N1*). In our study, detections of these 3 bacteria was 12%, 26%, and 33%. Also in the previous study, 56.4% of *S. pneumoniae* cases were accompanied by severe disease. Besides, in our research, 5 patients were hospitalized out of 7 with influenza A (*H1N1*) 2009 who had infection with* S. pneumoniae* with CT below 31 [28]. In a study conducted at Birmingham, UK [29] in 2009, 16% secondary bacterial infections were detected in children (10 out of 63 patients). In our study, detection of all three bacteria were 5%, 11%, and 10% in the control group, and comparable to a study performed by Normand et al. on the colonization rate of *S. pneumonia* in healthy individuals [30]. Other studies were done by Kassi Koon et al. [24] in the USA in 2009, evaluating viral and bacterial etiologic factors in 10,624 patients suspected of influenza infection. There, the proportion of patients carrying *S. aureus, S. pneumoniae, *and* H. influenzae *were 10%, 15%, and 3.5%, respectively for (*H1N1*) 2009 positive patients, and 12%, 8%, and 5% in *H1N1* (2009) negative patients [24]. In our study, the proportion of (*H1N1*) 2009 positive patients carrying 3 indicator bacteria were 12%, 26%, and 33%, and for *H1N1* negative individuals 9%, 2%, and 31%. Relatively small difference in percentage of people carrying three bacteria in two groups of *H1N1* positive and negative patients in both studies show that a new strain of swine influenza did not have much effect in favor of increasing carriers of target bacteria.

Statistical comparison between the two groups of (*H1N1*) 2009 and influenza A patients was not significant, thus indication that the pandemic influenza of 2009 occurred only mildly and the rate of secondary bacterial infection was the same as seasonal influenza that year. However, the number of individuals in the same group was small and therefore, the results need to be considered with some caution.

One of the causes of the decrease in rate of secondary infection was the experimental prescription of antibiotics to the patients in present and former studies and the other cause is the ability of the strain itself, which makes the pandemic. Pandemics spread by *H3N3* strain had higher mortality rates than *H1N1* or influenza B virus and also other causes are lack of PB1-F2 protein in the strain causing pandemic of 2009, low activity of neuraminidase and another reason is the medium immunity existed in the public [5], [9].

The reasons causing the appearance of difference percentage of bacterial secondary infections of influenza A (*H1N1*) in 2009 is condition of studies patients including chronic disease of the liver, lungs, or heart, or systemic metabolic disease such as diabetes. Therefore, single variable evaluations may cause higher rates of bacterial secondary infection [31], [32].

According to the risk of avian influenza pandemic, which is too pathogen, increase in age of populations and patients having immune deficiency, diabetes, hearts, lungs and kidney diseases, the risk of influenza and secondary bacterial infections must be assumed seriously. Using antibiotics for preventing the incidence of secondary infections does not seem to be prudent and causes induction and spreading of bacterial antibiotic resistance Using bacterial and viral vaccines such as *Haemophilus influenzae* type b and *pneumococcal* vaccines seems to be essential; besides injection of influenza vaccine is recommended for risky people.

## 5 Conclusion

Our study suggests that bacterial co-infection is not uncommon in *H1N1 *infected patients and laboratory investigations should go beyond establishing a viral cause alone. Bacterial co-infection was more frequently seen in the older age groups and was associated with higher rates of complications. Quantitative detection of secondary bacterial infection by QR-PCR can help us for distinguishing colonization from infection and controlling misuse of antibiotics and bacterial drug resistances. 

## Notes

### Competing interests

The authors declare that they have no competing interests.

### Acknowledgments

This article was sponsored in tuberculosis & lung research center and Tabriz University of Medical Sciences Center. We would like to thank Mr. Barzegar for excellent technical assistance.

## Figures and Tables

**Table 1:**
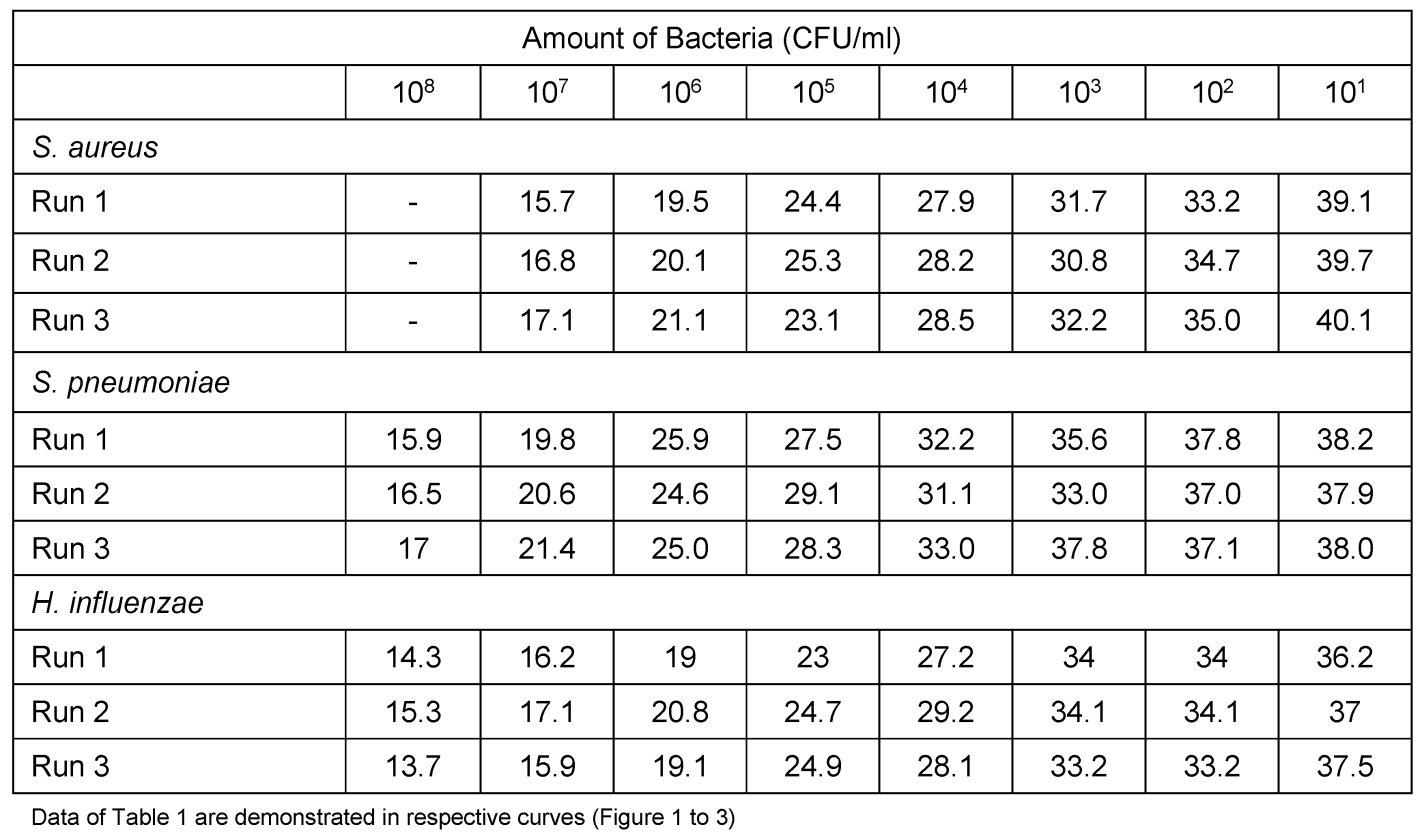
Interassay variation in cycle threshold (CT) values obtained with dilution series of the reference strains (percent variation)

**Table 2:**
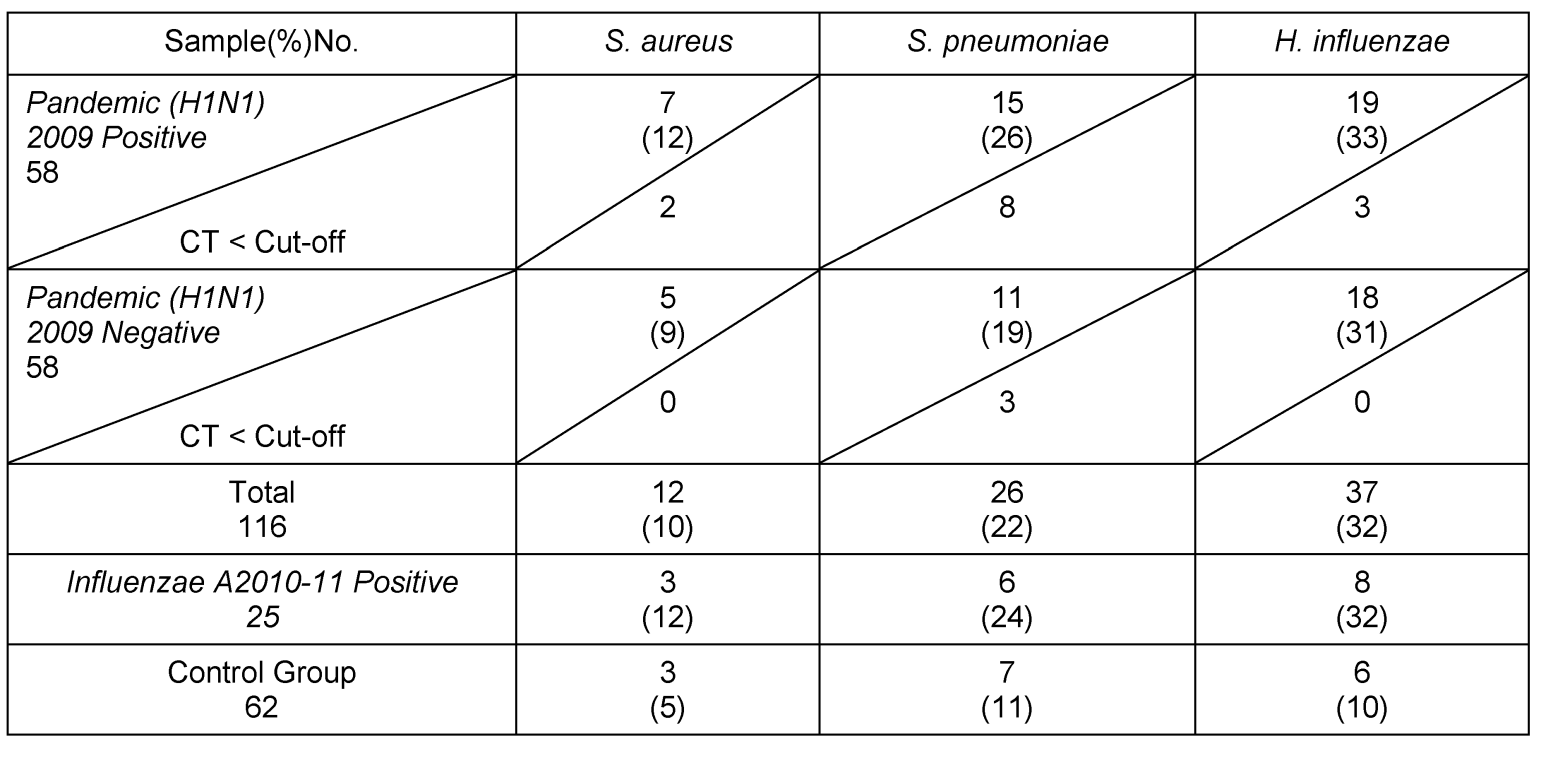
Results of screening of clinical samples for bacterial respiratory targets

**Table 3:**
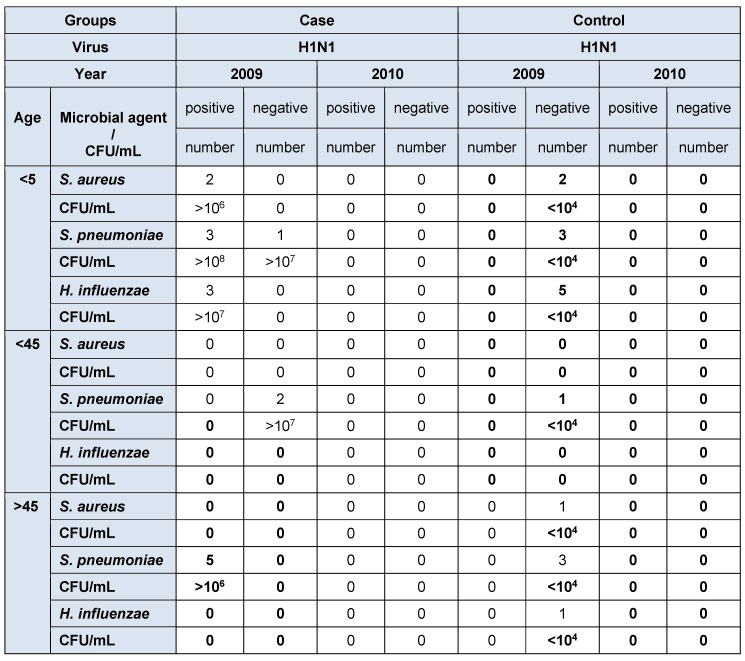
Identification and quantification real-time PCR result
